# Considerations on the Development of Therapeutics in Vascular Calcification

**DOI:** 10.3390/jcdd12060206

**Published:** 2025-05-29

**Authors:** Ana M. Valentin Cabrera, Sophie K. Ashbrook, Joshua D. Hutcheson

**Affiliations:** Department of Biomedical Engineering, Florida International University, Miami, FL 33174, USA; avale111@fiu.edu (A.M.V.C.); sashb010@fiu.edu (S.K.A.)

**Keywords:** vascular calcification, cardiovascular disease, chronic kidney disease, atherosclerosis, calcification paradox, extracellular vesicles, biomechanics, therapeutics

## Abstract

Cardiovascular disease is the leading cause of death worldwide. Vascular calcification, the deposition of calcium phosphate mineral in the arterial wall, is the most significant predictor of morbidity and mortality. Vascular calcification can present as either medial or intimal calcification. Medial calcification is most prevalent among patients with chronic kidney disease. Intimal calcification is associated with atherosclerosis and chronic inflammation. In both cases, vascular smooth muscle cells undergo osteogenic differentiation, leading to mineral deposition and associated wall stiffening; however, the effects on cardiovascular function and morbidity vary depending on mineral morphology and location. This review investigates vascular calcification, the mechanisms leading to calcium deposition, and what to consider when developing therapeutics for vascular calcification.

## 1. Introduction to the Development of Therapies in Vascular Calcification

### 1.1. Establishing a Need for Vascular Calcification Therapies

Vascular calcification represents the most significant predictor of cardiovascular events, with no current therapeutic options for prevention or treatment. Although once considered an aging, passive consequence, vascular calcification can also be due to active deposition mechanisms [1]. Vascular calcification significantly impacts different arteries, negatively impacting their function and increasing cardiovascular risk. Vascular calcification in the coronary arteries is a risk factor for myocardial infarction [2]. In the carotid and cerebral arteries, vascular calcification signifies an increased risk of stroke, peripheral arterial calcification is associated with peripheral artery disease, and renal calcification is associated with increased all-cause mortality risk [3,4,5]. With no forms of treatment available for vascular calcification, new mechanistic insight is needed to identify potential therapeutic strategies.

In addition, other clinical risk factors can contribute to the pathogenesis of vascular calcification, such as chronic kidney disease (CKD). CKD is defined as a group of abnormalities that can affect kidney structure or function for more than 3 months with health implications [6]. In dialysis patients, calcification is accelerated by hemodialysis and calcium-phosphate disorders associated with CKD [7,8]. In CKD patients not yet on dialysis, 50% have coronary artery disease, whereas 70–90% of dialysis patients exhibit significant coronary artery disease, suggesting the acceleration of cardiovascular remodeling in the later stages of CKD [9,10]. This review explores the underlying mechanisms of vascular calcification, the processes driving calcium deposition, and key considerations for the development of targeted therapeutics.

### 1.2. Active Mechanisms in the Development of Vascular Calcification

Vascular calcification used to be considered a degenerative process; however, in recent decades, it has been demonstrated to share mechanisms similar to bone remodeling mechanisms. Vascular smooth muscle cells (VSMCs) are contractile cells that reside in the medial layer of large arteries and can also migrate into the intima during vascular remodeling [11]. These cells maintain the vessel’s lumen size and tone, thus regulating blood pressure. VSMCs exhibit considerable phenotypic plasticity to maintain vascular homeostasis [12,13]. However, when local or systematic pressures persist, this leads to pathological remodeling [13]. In response to pathological stimuli, such as high levels of phosphate and calcium within arteries, VSMCs can undergo osteogenic differentiation and induce mineralization, mimicking the process of bone formation [11]. Osteogenic VSMCs release the specialized pro-calcific extracellular vesicles (EVs) responsible for calcification formation within the vascular wall [14].

EVs are lipid bilayer vesicles released by almost all cell types. EVs range between 50 and 1000 nanometers in size and are responsible for the intracellular and extracellular transportation of biological information in both health and disease [15,16]. Generally, EVs function to maintain intracellular and extracellular homeostasis. A class of EVs first reported in bone, known as matrix vesicles, contains calcium-binding proteins and enzymes that generate free phosphate [17]. These particular EVs then nucleate nascent minerals by promoting interactions between calcium and phosphate [18,19]. In pathological conditions, VSMCs release similar EVs, though their derivation appears different than the classical matrix vesicles reported in bone [20]. Unlike matrix vesicles from bone osteoblasts, intracellular trafficking processes load EVs from osteoblast-like VSMCs with calcification-promoting factors such as calcium-binding proteins, matrix-degrading enzymes, and microRNAs [14,18,21]. Once released from the cell, these EVs promote the nucleation and growth of minerals, and have been visualized using ultrastructural analyses in calcified human aortic valves, medial calcifications, and intimal atherosclerotic plaques [22,23].

### 1.3. Master Regulators of Vascular Smooth Muscle Cell Osteogenic Differentiation

#### 1.3.1. Wnt Signaling

The Wnt signaling cascade is associated with the osteogenic transition of VSMCs. Currently, 19 Wnt glycoproteins belong to the canonical or non-canonical Wnt pathway [12,24]. The canonical Wnt pathway controls bone formation and osteoblast differentiation, making it widely studied in vascular calcification [24]. The signaling cascade is activated by binding canonical Wnt ligands to a frizzled receptor associated with co-receptors on the plasma membrane-like lipoprotein-related peptide 5/6 (LRP5/6) [24,25]. Once the frizzled receptor is activated, disheveled (DVL) recruits the multiprotein destruction complex scaffolded by Axin and composed of glycogen synthase 3 (GSK-3), casein kinase 1, adenomatous polyposis coli (APC), and β-catenin [12]. In the absence of Wnt, Axin mediates the destruction of β-catenin. When Axin is recruited to the plasma membrane due to Wnt signaling, an accumulation of β-catenin occurs [25,26]. This, in turn, activates a cascade that promotes the nuclear translocation of β-catenin, activating the genes involved in cell proliferation and differentiation [27]. The Wnt pathway upregulates the genes associated with bone growth and VSMC osteogenic differentiation, including the master osteogenic transcription factor RUNX2, nuclear factor-κB ligand (RANKL), osteoprotegerin (OPG), and versican (VCAN) [12,28].

The canonical Wnt pathway has been investigated as a potential treatment for vascular calcification. Inhibitors such as full-length carboxypeptidase E (F-CPE) and sclerostin (SOST) can regulate proliferation and differentiation [12]. F-CPE combines with the frizzled receptor and the WNT3a ligand to form a complex that decreases the expression of β-catenin, halting the Wnt cascade [29]. Similarly, SOST binds to the LRP5/6 co-receptors, thus inhibiting RUNX2 and the progression of the Wnt pathway [30,31]. F-CPE in the context of vascular calcification has not been explored. Still, SOST could be a potential therapeutic target for preventing Wnt proteins from attaching to the frizzled and LRP 5/6 coreceptors [30].

#### 1.3.2. Bone Morphogenic Proteins

In addition to Wnt, bone morphogenic proteins (BMPs) are also associated with bone mineralization and have been observed in regions of vascular calcification. BMPs are a group of multifunctional cytokines within the transforming growth factor- β (TGF- β) superfamily known for their osteogenic properties and importance in VSMC differentiation [32,33]. BMP signaling leads to the upregulated expression of RUNX2, osteogenic differentiation of VSMCs, and vascular calcification.

The most studied BMPs in calcification are BMP2, -4, -6, and -7. BMP2, BMP4, and BMP6 are closely related and play a major role in cardiac arteriopathy and the stimulation of osteoregulatory genes leading to differentiation [32]. BMP2 is widely studied as a target for vascular calcification treatment and therapeutics. Studies have investigated the modulation of BMP2 with matrix Gla protein (MGP), the utilization of BMP inhibitor LDN-193189, increased vitamin K, the upregulation of Smad6, fetuin-A treatment, and inhibition of BMPs by Noggin [34,35,36,37,38,39]. However, when considering these therapeutic options to inhibit BMP2, as well as any other BMPs, the contradictory effects of decreasing BMP7 expression must be explored. A BMP7 deficiency increases osteogenic differentiation, but when abundant, a normal contractile VSMC phenotype is promoted [40]. This makes finding treatments challenging due to the need for lowering BMP2, -4, and -6 while leaving BMP7 unaltered and abundant. All these considerations are being further studied to understand the differential signaling in VSMCs that leads to osteogenic differentiation. These factors that lead to and control vascular calcification in disease-specific contexts will be valuable for the development of new therapeutics.

### 1.4. Comorbidities and the Pathophysiology of Vascular Calcification

#### 1.4.1. Intimal Versus Medial Calcification

The arterial wall consists of three layers: the tunica intima, tunica media, and tunica externa or adventitia. The tunica intima, or intimal layer, contains a simple squamous endothelium surrounded by a connective tissue basement membrane. The endothelium regulates the exchange of materials throughout the artery. The tunica media, or medial layer, is the thickest layer of arteries and contains VSMCs. The medial layer provides structural support and regulates blood pressure by changing the vessel diameter through vasoconstriction and vasodilation. The tunica externa, or adventitia, consists of connective tissue with varying collagen and elastic fibers, becoming less dense toward the outer surface of the arterial wall. The adventitia binds to the surrounding connective tissue to hold the vessel in place [41].

There are two types of vascular calcification: intimal and medial calcification. Medial calcification can occur without the lipid deposition associated with atherosclerosis and often occurs in patients with diabetes mellitus, renal disease, and hyperparathyroidism [42]. The widespread mineralization observed in medical calcification causes increased arterial stiffness, diastolic heart failure, poor blood flow perfusion, chronic ischemia, and left ventricular hypertrophy, but does not typically result in lumen obstruction [43]. Intimal calcification is associated with atherosclerosis and can play a major role in influencing plaque biomechanical stability depending on mineral morphology. Differences between calcification types must be considered as clinical management and diagnostic strategies are developed, because they lead to distinct clinical consequences [44].

#### 1.4.2. Medial Calcification

Medial calcification, also called Mönckeberg’s sclerosis, causes the stiffening of the vascular wall due to hydroxyapatite crystal deposition, which decreases vessel compliance, increases pulse pressure, and can result in left ventricular hypertrophy [45]. Calcification in this layer occurs along the elastic lamina and is often associated with diabetes mellitus and CKD [46]. Although patients do not experience blood flow obstruction from the presence of medical calcification, reduced vessel elasticity and compliance can lead to atherosclerosis and tissue hypoxia due to reduced perfusion [4].

Independent from atherosclerosis, medial calcification is associated with diabetes mellitus, CKD, and aging. Diabetic patients have multiple risk factors that predispose them to a high frequency of medial calcification, including inflammation, oxidative stress, adiposity, insulin resistance, and hyperphosphatemia [47]. CKD patients have advanced medial calcification, even before dialysis treatment, due to increased oxidative stress caused by hyperphosphatemia, calcium dysregulation, hyperparathyroidism, and uremia. End-stage renal failure leads to the dysregulation of serum phosphate and VSMCs exposed to high levels of phosphate in the blood undergo osteogenic differentiation. Both calcium and phosphate contribute to upregulating osteogenic markers such as RUNX2, alkaline phosphatase, and osteopontin, leading to medial calcification. Increased parathyroid hormone—hyperparathyroidism—is also common in CKD patients, stimulating the renin–angiotensin–aldosterone and sympathetic nervous systems and increasing arterial blood pressure. Uremia has also been found to induce the expression of osteogenic proteins and osteoblast differentiation factor core-binding factor alpha-1, contributing to the pathological remodeling of VSMCs [44]. With all of these factors combined, the prevalence of vascular calcification increases in patients with CKD.

#### 1.4.3. Intimal Calcification

Intimal calcification is an inflammation-mediated pathology in the tunica intima during late-stage atherosclerosis. Stress induced by modified lipoproteins and cytokines induces osteogenic differentiation in VSMCs, leading to calcification deposition in atherosclerotic plaques. Vascular calcification may contribute to plaque destabilization, which can lead to heart attack, stroke, or edema as the blood flow is disrupted by plaque rupture and thrombus formation. Factors such as hypertension, hypercholesterolemia, diabetes, and smoking increase the risk for atherosclerotic plaque development and progression. The presence and extent of calcification through a calcium score is currently one of the best predictors of cardiovascular morbidity and mortality, and studies have demonstrated a positive association with calcium score and all-cause mortality [48].

Atherogenesis—the formation of atherosclerotic plaques—begins when lipids, primarily oxidized low-density lipoproteins (LDLs), circulating in the bloodstream accumulate in the artery wall [49]. Positively charged lipoproteins stick to negatively charged intimal lining proteoglycans [48]. These regions of fatty material can become plaques or atheromas. The accumulated fat is separate from the vessel lumen by a fibrous collagen cap and causes the thinning of the medial layer, allowing VSMCs to migrate into the intima from the media [48].

Endothelial cells and VSMCs begin to release inflammatory cytokines after the initiation of hypercholesteremia, such as monocyte chemotactic protein-1 and interferon-inducible protein-10, and adhesion molecules, such as vascular cell adhesion molecules. The chemokines and adhesion molecules recruit monocytes and T cells, resulting in an inflammatory cascade. Monocytes differentiate into macrophages in the presence of macrophage colony-stimulating factor, with the upregulation of scavenger and toll-like receptors, leading to the intracellular accumulation of cholesterol via macrophages and the development of foam cells [50,51]. Studies have shown a great diversity of immune cells in atherosclerotic plaques. Carotid artery atherosclerotic plaques of patients without recent stroke or transient ischemic attack have activated T cells and macrophages with interleukin β (IL-1β) signaling [52]. Macrophages themselves can also release EVs that nucleate minerals [53].

VSMCs in the atheroma also differentiate into various phenotypes, such as fibroblasts, foam cells, and osteogenic cells, in response to the stress caused by the atheroma. [46,54] The osteogenic cells are responsible for releasing calcifying extracellular vesicles into the atherosclerotic plaque within the intimal layer of the artery [55]. Necrotic cell death occurs within advanced plaques due to the increased cell volume, organelle swelling, and chromatin condensation that causes the cell membrane to rupture. These dead cells and their inner contents accumulate, forming a necrotic core filled with cellular debris [51,56]. Cell death in the necrotic core has been shown to lead to mineral nucleation within the atherosclerotic plaque [57]. However, as in medial calcification, VSMCs have been shown to play a role in nucleation through osteogenic differentiation downstream of elevated inflammation. This mineralization may serve as an endpoint for plaque remodeling [53]. However, maladaptive remodeling during the progression of atherosclerotic plaque can indicate the destabilization of the plaque, leading to adverse outcomes.

#### 1.4.4. Genetic Predisposition to Ectopic Calcification

In addition to these comorbidities that lead to vascular calcification, some individuals develop ectopic calcification due to genetic mutations. For example, arterial calcification due to CD73 deficiency (ACDC) is a genetic disease that results in the vascular calcification of the artery medial layer [58]. CD73 converts extracellular adenosine monophosphate and pyrophosphate (PPi), a potent calcification inhibitor, into adenosine and inorganic phosphate (Pi). CD73 deficiency reduces extracellular adenosine and increases tissue non-specific alkaline phosphatase (TNAP) activity, leading to an imbalanced PPi/Pi ratio driving vascular calcification. Patients with ACDC cannot break down extracellular AMP into adenosine and inorganic phosphate, causing progressive lower extremity calcification and limb ischemia. Cultured pluripotent stem cell-derived mesenchymal stromal cells from ACDC patients have increased TNAP activity in osteogenic conditions and decreased PPi [58]. Potential therapeutic targets for ACDC include adenosine receptor agonist, rapamycin, and etidronate.

Adenosine regulates vascular homeostasis by either coupling with G-protein-coupled receptors to inhibit the cAMP pathway (adenosine receptors A1 and A3) or activating cAMP pathways by coupling with Gα proteins (adenosine receptors A2a and A2b). Mice injected with ACDC iPSCs treated with A2b adenosine receptor agonist have significantly reduced calcification in teratomas derived from the iPSCs, likely due to the increased adenosine receptor signaling leading to decreased TNAP, thus increasing PPi. Mammalian target of rapamycin (mTOR) is a protein responsible for regulating cell proliferation, autophagy, and apoptosis [59]. mTOR was also found to mediate TNAP. The mTOR inhibitor rapamycin also decreased TNAP activity in ACDC patients’ primary fibroblast. Rapamycin also significantly reduced calcification in ACDC iPSC-derived teratomas in mice.

Etidronate, a bisphosphonate successfully used to treat generalized arterial calcification in infancy, has displayed effectiveness in slowing the progression of vascular calcification in ACDC patients [60], although the reversal of existing vascular calcification has not been successful. In vitro, etidronate was able to resolve existing calcified deposits in human mesenchymal stromal cells [61]. In vivo, mice with ACDC iPSC-derived teratomas showed significantly decreased calcification [58]. A pilot study of seven ACDC patients receiving etidronate for three years demonstrated trends indicating the slowing of vascular calcification progression when comparing computed tomography (CT) calcium scores and the ankle brachial index, markers of peripheral artery disease [60].

## 2. Common Models for Target Discovery

**Figure 1:**
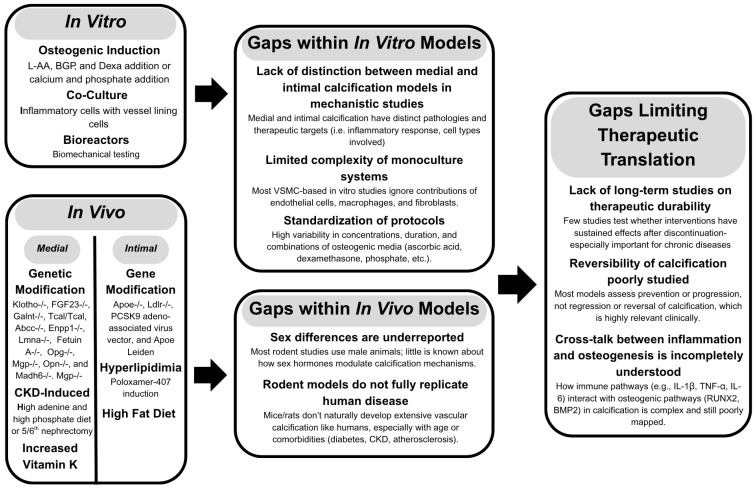
Overview of current in vitro and in vivo models of vascular calcification and key limitations in therapeutic development. In vitro systems commonly use osteogenic induction, co-culture models, and bioreactors, while in vivo models employ gene modifications, high-fat diets, and interventions such as vitamin K supplementation. Notable gaps include poor distinction between medial and intimal calcification, limited complexity in cell systems, variability in protocols, underrepresentation of sex differences, and limited translatability of rodent models. Therapeutic development is hindered by a lack of long-term studies, insufficient data on reversibility, and incomplete understanding of inflammatory-osteogenic crosstalk, all of which present critical barriers to clinical translation.

Identifying potential therapeutic targets for different types of calcification requires consistent models and the standardization of assays and reporting across the field (Figure 1). Despite the number of available models for vascular calcification, the complex mechanisms remain to be elucidated. Many models are not representative of clinically relevant calcification. Studies investigating vascular calcification must ensure differentiation between medial and intimal calcification. The generalization of vascular calcification should be avoided to ensure that the appropriate considerations on the development of therapeutics are made.

### 2.1. In Vitro Studies

Inducing osteogenic differentiation in vascular smooth muscle cells is a standard model for studying vascular calcification in vitro. VSMCs are commonly cultured with phosphate to mimic hyperphosphatemia [62]. Human VSMCs supplemented with calcium and phosphate have increased dose-dependent and time-dependent calcium deposition. Granular deposits can be found on the surface of these cultures, and increased collagen formation mimics ectopic calcification deposition.

Another common way to induce osteogenic differentiation in vitro is by supplementing cells with varying concentrations of ascorbic acid, β-glycerophosphate, dexamethasone, insulin, calciferol, calcium chloride, and sodium pyruvate [63]. Ascorbic acid stimulates the differentiation of vascular smooth muscle cells [64,65]. Ascorbic acid is also an essential cofactor in the hydroxylation of proline and lysine to form the essential amino acids for collagen biosynthesis [66]. Enhanced RUNX2 activity from ascorbic acid also induces alkaline phosphatase activity and the expression of bone matrix protein genes in vitro [67,68]. β-glycerophosphate acts as a substrate for alkaline phosphatase, serving as a phosphate source for mineralization [69]. Dexamethasone increases calcification in a dose-dependent and time-dependent manner [70]. It induces RUNX2 expression and enhances alkaline phosphatase activity, procollagen production, and cAMP activity [70]. Studies have demonstrated that dexamethasone, β-glycerophosphate, and ascorbic acid are required for the osteogenic differentiation of VSMCs in vitro. Multicellular platforms are currently being developed to further study intercellular interactions (e.g., endothelial cells co-cultured with VSMCs) in vascular calcification [71]. Bioreactors and dynamic culture systems are also used to replicate the relevant cardiovascular biomechanical stresses [72,73].

### 2.2. In Vivo Studies

While in vitro models can provide mechanistic insight by isolating specific factors or processes of pathogenesis, the closer recapitulation of complex intercellular interactions and systemic pressures still requires in vivo models. Due to the chronic nature of vascular calcification, mouse and rat models are often used due to their shortened lifespan, ease of genetic manipulation, and rapid reproduction rate. A major limitation of rodents in vascular calcification research is that they do not naturally develop significant vascular calcification at ages routinely used in studies. Many rodent models require manipulation for calcification to occur. However, they still offer valuable insight into the physiological environment that cannot currently be implemented in vitro [63].

#### 2.2.1. Medial Calcification

Medial calcification can be induced in mouse models through phosphate or pyrophosphate metabolism modification, including *Klotho^-/-^*, *FGF23^-/-^*, *Galnt^-/-^*, *Tcal/Tcal*, *Abcc^-/-^*, *Enpp1^-/-^*, and *Lmna^-/-^*. Most of the mice with phosphate or pyrophosphate metabolism modifications have growth retardation, reduced life spans, and fertility limitations. These mice often have aortic calcification, ectopic calcification, and hyperphosphatemia. Osteogenic signaling modifications can also be performed, including *Fetuin A^-/-^*, *Opg^-/-^*, *Mgp^-/-^*, *Opn^-/-^*, and *Madh6^-/-^*. *Mgp^-/-^* causes severe changes in mice after two weeks of age; these mice are smaller, with an increased heart rate, and aortic rupture is often fatal after two months. Other osteogenic signaling modifications lead to healthy-appearing phenotypes if the mice survive past weening. These mice have calcification in the media of the aorta and renal arteries and may have ectopic calcification [63,74].

Rather than modulating the proteins involved in the mineralization process, many models of medial calcification do so through the induction of renal dysfunction. The main ways to induce CKD-mediated vascular calcification in rodents are surgical, diet-based, and genetic modifications [74]. There are three genetically modified models of vascular calcification, including DBA2, LPK Disease, and CY+, with autosomal dominant PKD. These rodents have renal dysfunction, which makes them more prone to vascular calcification. Nephrectomy, which reduces the total kidney mass by five-sixths, can also be used in animals to induce CKD. Removal of two-thirds of the first kidney is followed by total nephrectomy of the second kidney. These mice have high serum creatinine and phosphorus and significantly decreased weight. These animals sometimes also require a high-phosphorus diet or calcitriol for at least 12 weeks to induce medial calcification. This model is limited in use because of the extensive surgical procedure, leading to increased death rates in these animals. Some animals are also resistant to severe kidney damage and have variable calcification, with most not calcifying before 12 weeks on the high-phosphate diet [75]. Diet and substance modifications can also be used independently to induce vascular calcification. Adenine and phosphate are often used in conjunction to produce CKD-induced medial calcification. Adenine supplementation in the diet mimics chronic uremia, leading to medial calcification. These rodents have increased uremia blood markers, renal fibrosis, and elastin disorganization as early as six weeks after starting the diet [76].

Vitamin D can be administered over three days, causing extensive medial calcification one week after injection. Withdrawal of the treatment causes the resorption of calcium deposits. Histological analysis of the aorta in rats given high dosages of vitamin D showed monocytes on the endothelium and macrophages with phagocytosed mineral particles [77]. These animals have increased calcium and phosphate levels, but the mechanism of action is not well known and may have anti-inflammatory effects aside from medial calcification [78]. VSMCs express vitamin D receptors; vitamin D administration stimulates calcium influx into the cells and calcification proteins such as alkaline phosphatase and osteopontin in vitro [79].

#### 2.2.2. Intimal Calcification

Genetic modifications to induce hyperlipidemia are the most common murine models to study atherosclerosis, and are often used with specialized diets and substances, such as poloxamer-407 (P-407) to induce vascular calcification in rodents [74]. P-407 is a surfactant block copolymer that causes dose-dependent hyperlipidemia by inhibiting lipoprotein lipase (LPL), which is needed to degrade triglycerides into fatty acids [80,81,82]. Mice develop extensive hyperlipidemia for 72 h after intraperitoneal injection of P-407. Intimal calcification is induced through lipoprotein gene modifications, including *Apoe^-/-^*, *Ldlr^-/-^*, and Apoe Leiden [63]. These mice demonstrate increased cholesterol levels and develop calcified atherosclerotic plaques after approximately 20 weeks on a regimen designed to promote plaque development. Oftentimes, these mice require a high-fat diet to develop significant vascular calcification within this period, despite the disruption of lipid metabolism. An abundance of genetic modifications can be combined with diets and substances to induce vascular calcification in vivo; the selection of the models is based on the experimental needs of the researcher.

*Ldlr^-/-^* mice have hindered LDL clearance from their blood plasma, leading to calcification in the aorta. The PCSK9 adeno-associated virus vector can induce calcification in C57BL/6 mice comparably to *Ldlr^-/-^* mice [83]. The viral vector is administered once; the mice are then placed on a high-fat diet for 20 weeks and sustain high cholesterol and similar atherosclerotic lesions. *Apoe^-/-^* also causes an inability to clear LDL, causing an accumulation of cholesterol that leads to atherosclerosis and intimal calcification. These mice develop extensive calcification in the aortic arch.

## 3. Considerations for Therapeutic Development

### 3.1. Modulating Mineral Formation

#### 3.1.1. Bisphosphonates

Bisphosphonates are widely used therapies in osteoporosis by inhibiting bone resorption by binding to hydroxyapatite [46]. Because of the high association between osteoporosis and vascular calcification, bisphosphonates were viewed as a potential therapeutics to reduce mineral formation, which could be a viable treatment option for patients with chronic kidney disease. In vivo and in vitro, bisphosphonates have been reported to suppress atherosclerosis and mineralization [84,85,86,87]. However, researchers have shown that bisphosphonates can cause inflammation and plaque rupture [88]. Clinically, bisphosphonates show differing outcomes from those presented in vitro and in vivo. Bisphosphonates such as alendronate and ibandronate show inconsistent results on the degradation of minerals, while etidronate limits further calcification [89,90]. Denosumab, a bisphosphonate being clinically tested, shows contradictory results, indicating in some cases that vascular calcification treatment is effective in osteoporotic patients, while in other studies, denosumab does not affect the progression of mineralization during a 3-year observation in postmenopausal women with osteoporosis [91,92,93]. Contradictory results with denosumab may be attributed to the divergent mechanisms between bone and vascular mineralization, known as the calcification paradox. Many other factors may contribute to the contradictory results, including the timing of treatment, the pathology being studied, and the type of bisphosphonate used. This demonstrates the clinical inconsistency of bisphosphonates and indicates the need for further research and long-term clinical studies on their ability to decrease mineralization.

#### 3.1.2. Phosphate Binders

Similarly to bisphosphonates, phosphate binders have been studied as a form of treatment for calcification. Specifically in CKD patients, there is a major mineral and bone disorder due to the imbalance of promotors (phosphate and calcium) and inhibitors (magnesium, fetuin-A, and bicarbonate), which makes this population more susceptible to vascular calcification [94,95,96,97,98,99]. Because of this large influx of phosphate, phosphate binders are prescribed medications that help to lower phosphate levels and slow the progression of vascular calcification. Commonly prescribed phosphate binders are sevelamer and calcium acetate [100]. Both sevelamer and calcium acetate bind to phosphate to lower serum phosphorous. In addition, calcium acetate absorbs calcium, causing an influx where sevelamer showed little evidence of disease development in patients that started with no coronary calcification [93,94]. Clinical studies have shown a decrease in vascular calcification minerals due to aluminum–calcium-free iron-based phosphate binders, tenapanor phosphate binders, and magnesium carbonate phosphate binders [95,96,97]. Calcium-free phosphate binders serve as a promising target for the treatment of vascular calcification.

#### 3.1.3. Tissue Non-Specific Alkaline Phosphatase

TNAP is a ubiquitous enzyme found mainly in mineralizing tissues, kidneys, and the central nervous system [101,102]. TNAP regulates PPi by hydrolyzing PPi to Pi, increasing Pi and vascular calcification. PPi is a potent inhibitor of calcification by binding to hydroxyapatite crystals, preventing further mineralization. The balance of calcification inhibitors like PPi and promoters such as calcium and phosphate is essential for the calcification of bones and teeth, while preventing the pathological calcification of soft tissue [103]. The upregulation of TNAP in VSMCs and endothelial cells has been found in many pathological conditions, including CKD, diabetes, and obesity [104,105]. Epidemiological studies have demonstrated that higher TNAP levels in serum are significantly associated with an increased risk of cardiovascular disease in both men and women [106]. The inhibition of TNAP has surfaced as a new therapeutic target for vascular calcification.

Several TNAP inhibitors have been formulated or analyzed through computational models. The first TNAP inhibitors studied, L-homoarginine and levamisole, had poor binding capacity and non-specific binding. Several aryl sulfonamides were found to be selective inhibitors of TNAP, and the oral administration of one inhibitor in particular, SBI-425, demonstrated the inhibition of plasma TNAP activity and arterial calcification in mice with endothelial or vascular smooth muscle cell overexpression of TNAP [107]. Rats with warfarin-induced vascular calcification also had reduced vascular calcification when given SBI-425. SBI-425 was also tested on atherosclerotic plaque calcification in *Apoe^-/-^* mice fed a high-fat diet [108]. The TNAP inhibitor prevented calcification, reduced cholesterol and triglyceride levels in the blood, and protected mice from atherosclerosis without impacting bone mineralization. Computational studies of highly selective TNAP inhibitors have also displayed high potency, representing potential new compounds for the treatment of vascular calcification through TNAP inhibition [109].

#### 3.1.4. Vitamin K

Vitamin K is required for MGP activation as a calcification inhibitor [107]. For MGP to inhibit calcification, vitamin K is required as a cofactor in the carboxylation of the protein. Mice that are deficient in MGP die within two months due to blood vessel rupture caused by vascular calcification and other soft tissue pathological calcification [110]. Warfarin, a commonly prescribed anticoagulant, induces vascular calcification in rats by antagonizing the vitamin-K-dependent carboxylation of MGP. This calcification can be reversed by giving rats a diet rich in vitamin K [111]. Observational studies based on dietary intake suggest that vitamin K2 may protect against vascular calcification over vitamin K1 [112]. A review of 14 randomized controlled trials determined that vitamin K supplementation significantly slowed the progression of coronary artery calcification scores. The difference in outcomes between genetic disruption and the pharmacological inhibition of MGP may be attributed to the timing and reversibility of MGP inactivation. Complete genetic knockout results in a total, lifelong absence of functional MGP, starting from development, leading to severe and fatal calcification, which is not applicable in humans. In contrast, pharmacological inhibition with warfarin causes a partial reduction in MGP activity, where dietary vitamin K availability can mitigate the extent of calcification, thus explaining the less severe outcome observed with pharmacological inhibition. Despite these results, more rigorous testing is needed to validate that vitamin K supplementation is efficacious in treating vascular calcification.

#### 3.1.5. Receptor for Advanced Glycation End Products

Advanced glycation end products (AGEs) and the receptor for advanced glycation end products (RAGEs) are known to play a role in vascular calcification, particularly in patients with diabetes mellitus. Patients with diabetes have an increased risk of vascular calcification due to the increase in oxidative stress, hyperglycemia, hyperkalemia, and hypercalcemia [113,114,115]. These risk factors also increase AGEs, causing increased expression and activation of RAGEs, leading to an increase in oxidative stress through elevated reactive oxygen species and NADPH oxidase, as well as a decrease in endothelial nitric oxide synthase. RAGEs also induce an increase in inflammatory cytokines, including TNFα [116]. Combined, these effects lead to endothelial dysfunction, atherosclerosis, and subsequently vascular calcification. *Apoe^-/-^* mice that lack RAGEs have decreased atherosclerosis [117]. *Apoe^-/-^* mice stimulated by proinflammatory cytokine S100A12, which activates RAGEs, have an increased atherosclerotic plaque size and calcified plaque area [118]. Another study further elucidates this mechanism by demonstrating that under hyperglycemic conditions, the S100A9-RAGE axis in macrophages promotes the secretion of EVs with high calcific potential [119]. These EVs contribute to the formation of microcalcification within atherosclerotic plaques. In diabetic Apoe−/− mice, genetic deletion or siRNA silencing of S100A9 significantly reduced vascular inflammation and calcification. Moreover, human carotid plaques from diabetic patients showed elevated S100A9 and RAGE expression, correlating with osteogenic activity and microcalcification, further demonstrating the RAGE pathway as a key contributor to diabetes-associated vascular calcification. Glycomimetics, molecules that mimic heparan sulfate, have demonstrated efficacy in reducing calcification in vitro in β-glycerophosphate-induced vascular calcification in VSMCs [120]. VSMCs supplemented with serum from patients with critical limb ischemia had significantly decreased calcification in vitro through the attenuation of the RAGE pathway [121]. Inhibiting the RAGE pathway highlights the crucial role of regulating mineral formation by reducing oxidative stress, as well as controlling inflammation through the modulation of inflammatory cytokines.

### 3.2. Modulating Inflammation

#### 3.2.1. Tumor Necrosis Factor-α

Targeting inflammation within atherosclerosis may improve patient outcomes beyond the lipid-lowering interventions currently used. Tumor necrosis factor-α (TNFα) is an inflammatory cytokine that leads to necrosis or apoptosis, induces inflammatory gene expression, and has been found in atherosclerotic plaques [122]. Plasma TNFα in humans predicts incident myocardial infarction, though the mechanism for the proatherogenic role of TNFα is unclear [123]. Deficiency in TNF receptor-1 reduced atherosclerotic plaque lesion size two-fold in *Apoe^-/-^* mice [124,125]. TNFα promotes atherosclerosis through increased LDL transcytosis via endothelial cells, activating transcription factors nuclear factor kappa B and peroxisome proliferator-activated receptor γ (PPARγ) [126]. TNFα treatment induced osteogenic differentiation in bovine aortic smooth muscle cells, increased the expression of TNAP, and elevated intracellular cAMP [127]. TNFα stimulated TNAP expression and activity in human vascular smooth muscle cells by inhibiting PPARγ. M1 macrophages also secrete TNFα to stimulate vascular calcification in VSMCs [128].

While targeting this inflammatory cytokine through the genetic disruption of TNFα decreases atherosclerosis in mice, the pharmacological inhibition of TNFα through the administration of anti-TNFα monoclonal antibody in *Ldlr^-/-^* mice increases the atherosclerotic plaque burden [129].

#### 3.2.2. IL-1β

IL-1β is another proinflammatory cytokine that activates TNAP and is secreted by atherosclerotic plaques with vascular calcification [130,131]. It is secreted by macrophages, fibroblasts, and VSMCs, among others, and induces VSMCs’ osteogenic differentiation [130,132]. IL-1β has been implicated in various cardiovascular diseases, including hypertension and coronary artery disease [133,134,135]. Monoclonal antibodies against IL-1β have been tested to determine the potential for therapeutics targeting inflammation in atherosclerosis. *Ldlr^-/-^* mice on a high-fat diet treated with a monoclonal antibody against IL-1β had significantly reduced vascular calcification [136]. In the Canakinumab Anti-inflammatory Thrombosis Outcome Study (CANTOS), an IL-1β inhibitor, canakinumab, significantly reduced the rates of cardiovascular events and heart failure hospitalization in patients with a history of myocardial infarction [137,138,139]. The CANTOS trial demonstrated the benefits of targeting inflammation in atherosclerosis, but there was no effect on cardiovascular mortality, and it was associated with increased mortality due to infection. These findings suggest that IL-1β contributes to vascular calcification and plaque instability by promoting inflammation and VSMC osteogenic differentiation. Inhibition reduces inflammation and stabilizes plaques; however, no mortality benefit was observed, likely due to irreversible disease in many patients, and increased infection risk reflects IL-1β’s role in host immune defense.

### 3.3. Calcification Paradox

When developing therapeutics for calcification, off-target effects on bone are an important consideration. Vascular calcification mimics bone formation. However, vascular mineralization is commonly associated with an observed decrease in bone mass [140,141]. This contradictory effect is known as the calcification paradox. Although osteogenically differentiated VSMCs and bone cells show similar endpoint mineralization, the pathways and mechanisms associated with each tissue differ.

As previously discussed, VSMCs release calcifying EVs, which mineralize calcification in bone; however, mineralization depends on the secretion of MVs from osteoblasts, chondrocytes, odontoblasts, tenocytes, and cementoblasts [142]. MVs, unlike EVs, bud directly from the membrane into the extracellular environment [143]. MVs aggregate calcium and phosphate to nucleate the hydroxyapatite mineral observed in bone [144]. Bone and VSMC mineralization, although alike in expression of BMP2 and RUNX2, differ in mineral type and quality [18,145]. Clinical studies note the inverse correlation between bone mineral density and arterial minerals, suggesting an imbalanced phosphate/calcium content promoting the simultaneous remodeling of both tissues [146,147,148]. Additionally, it has been hypothesized that systemic inflammation promotes a decrease in bone mineral density while increasing calcification. However, recent studies show that altered protein dynamics result in divergent mineralization responses [149,150]. The divergence in cellular mechanisms may present an opportunity to target pathophysiological processes in vascular calcification while simultaneously promoting bone health.

### 3.4. Micro- vs. Macrocalcifications

#### 3.4.1. Macrocalcifications

Vascular calcification grows from initial mineral nucleation in single EVs (~100 nm) to mature macrocalcifications of over 50 μm [151]. In medial calcification, macrocalcifications can span the entire circumference of the artery, severely inhibiting the peristaltic ability of the vessel to pump blood through the body, leading to left ventricular afterload and decreased peripheral artery perfusion [43]. Macrocalcifications are also often indicative of stable plaques. Atherosclerotic plaque rupture occurs when local mechanical stress leads to the failure of the fibrous cap that covers the plaque. Macrocalcifications have lower overall stress due to their higher load-bearing capacity, making large, calcified atheromas more stable than microcalcifications and easier to image [152,153].

Relatively large macrocalcifications are detectable via common imaging modalities. CT scans possess high spatial and temporal resolution, which provides detailed information on plaque morphology non-invasively [154,155,156]. Currently, CT is the most common imaging technique for coronary artery disease due to its low cost and non-invasive nature [157]. Magnetic resonance imaging (MRI), like CT scans, is non-invasive in imaging calcification with a lack of ionizing radiation [158]. Using a multi-pulse sequence that outlines the location and volume, MRIs are superior in soft tissue resolution, but still suffer from the poor temporal resolution, which causes difficulties in visualizing complex motions caused by cardiac contractions and respiration [159]. MRIs and CT scans provide information on macrocalcifications’ density, size, and spatial distribution.

Statins, lipid-lowering medications given to patients at high risk of cardiovascular disease, often cause increased CAC scores and a decreased risk of cardiac events [160]. This suggests that statins could increase plaque density, thus increasing plaque stability. CAC has been a non-invasive diagnostic marker for atherosclerosis since the 1940s, based on the extent of calcium in CT scans. Patients with a moderate to high Agatston CAC score have been correlated with a significant risk of having cardiovascular events in the near future [161]. Statins have been shown to increase calcification, likely due to the formation of macrocalcification that can be locally stabilizing and reduce the risk of any individual plaque from rupturing [162]. However, altered hemodynamics due to plaque presence can promote additional plaque formation, and statins do not reverse existing late-stage plaques [163,164]. This may explain the observation that significant residual risk remains in patients taking statins. In the general population, high CAC scores indicate more plaque, increasing the likelihood of the presence of a vulnerable plaque that may be prone to rupture.

#### 3.4.2. Microcalcifications

Microcalcifications, which are calcium deposits with diameters ranging from 1 μm to 50 μm, form within atheromas and serve as precursors to larger macrocalcifications [165]. These microcalcifications significantly increase stress within the fibrous cap, contributing to plaque instability and rupture [166,167]. Studies have demonstrated that microcalcifications amplify focal stress in the fibrous cap by approximately twofold, leading to interfacial debonding, weakening the cap, and ultimately promoting plaque rupture [168,169,170]. Theoretical models have further explored the possibility that cavitation, rather than interfacial debonding, may initiate fibrous cap rupture [171]. Rupture occurs at high-stress microcalcification sites when the tensile stress exceeds a critical threshold, triggering the expansion of tiny bubbles at points of tension [172]. These models illustrate that stress magnitude increases with larger microcalcification diameters and decreases as the distance between calcified regions increases. Findings from these investigations highlight microcalcifications’ critical role in destabilizing plaques, emphasizing how their size and spatial distribution can influence plaque vulnerability. These studies underscore the pathogenic role of microcalcifications in inflammation-driven atherosclerosis and highlight the importance of addressing these sub-micrometer deposits in the context of plaque stability and rupture risk.

Microcalcifications are clinically more challenging to image due to their small size, and cannot be detected with the spatial resolution of CT and MRI [173]. In addition, CT and MRI give no information on inflammatory activity in plaques, creating a need for more advanced imaging techniques. Positron emission tomography (PET) uses non-invasive techniques to image the vessel wall, specifically microcalcifications, with ^18^F-sodium fluoride (^18^F-NaF), a highly specific and sensitive bone deposition tracer [174]. Specifically, ^18^F-NaF PET differentiates microcalcifications due to the strong affinity of the radioligand with newly formed hydroxyapatites that are not present in stable, old crystals found in macrocalcifications, which is explained by the high surface area to volume ratio of hydroxyapatite [175]. In addition to ^18^F-NaF, ^18^F-fluorodeoxyglucose (^18^F-FDG) can also be administered as a macrophage and inflammation marker, providing a complimentary atherosclerosis assessment [154]. Although they are novel methods for non-invasive microcalcification imaging and inflammation tracing, ^18^F-NaF and ^18^F-FDG PET have a lower spatial resolution, and due to continuous cardiac movement, accurate quantification of coronary artery microcalcifications can be hard to obtain.

Though more invasive than the techniques described above, intravascular ultrasound allows for the high-resolution identification of plaque features; however, this technique has limited axial resolution, and microcalcifications are undetectable [176]. Unlike intravascular ultrasounds, optical coherence tomography (OCT) assesses superficial calcium, including the length, arc, thickness, and volume [177]. OCT’s high spatial resolution could provide a new microcalcification imaging technique. However, OCT has limited tissue penetration (1–3 mm), which complicates the visualization of the plaque in addition to being an invasive form of imaging [154].

### 3.5. Timing of Treatment

The treatment of calcification should depend on the type present. In intimal calcification, the stabilization of plaque may be a short-term goal, but ideal therapeutics would return the vessel to a normal, healthy state. By the time calcifications are typically diagnosed, they are identified as stable macrocalcifications or at the endpoint of the process. Although many studies demonstrate the ability to prevent vascular calcification, reversibility remains unclear.

Resorption occurs through osteoclasts, which remove mineral and organic components when investigating bone inflammation [178]. Osteoclasts possess monocyte and macrophage origins, which are prevalent cells in the proinflammatory response during atherosclerosis [179]. Within atherosclerotic plaques, it has been observed that macrophages can develop into osteoclast-like cells, but are unable to resorb calcification minerals [180,181]. While these osteoclast-like macrophages in plaque could provide a unique therapeutic potential, this raises a concern regarding whether it could be beneficial or detrimental to reverse calcification. If stable plaques and macrocalcifications are regressed, this would create new, unstable microcalcifications. More research regarding the potential therapeutic reversal of minerals should be explored.

In medial calcification, reversal therapy may not be feasible without the presence of inflammatory cells that help resorb the mineral. CKD patients are the most prone to medial calcification due to the high phosphate imbalance. CKD patients with no detectable calcification have 8-year-all-cause survival rates of 90%, compared to 50% survivability in age-matched patients with medial calcification [8,182]. Because there is a correlation between medial calcification and beginning dialysis, preventative therapies should be investigated for patients at the start of dialysis. Given the varying timelines for effective treatment in intimal and medial calcification cases, it is imperative to employ distinct calcification models depending on the underlying pathology. Due to the fact that CKD patients are easily identifiable and develop calcification rapidly, they may be well suited for first-in-man clinical trials, which could then be generalized to others in need of therapeutics for vascular calcification.

## 4. Conclusions and Future Directions

### 4.1. Summary of Key Findings

**Figure 2:**
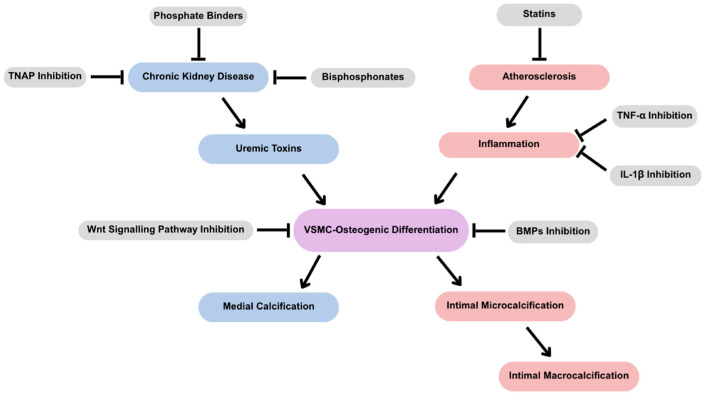
Pathophysiological mechanisms and potential therapeutic targets in vascular calcification. VSMC osteogenic differentiation, driven by BMPs and Wnt signaling pathways, leads to medial and intimal calcification. Medial calcification is often associated with CKD, which may be addressed through TNAP inhibitors, phosphate binders, and bisphosphonates. Intimal calcification, linked to inflammation (e.g., TNF-α and IL-1β) and atherosclerotic plaque development, may be mitigated by anti-inflammatory agents and vitamin K supplementation. This schematic highlights key molecular pathways and therapeutic interventions relevant to vascular calcification.

Vascular calcification is now understood as an active, cell-regulated process rather than a passive consequence of aging (Figure 2). Pathological conditions such as CKD and atherosclerosis lead to the osteogenic differentiation of VSMCs, driven by phosphate and calcium, inflammation, and oxidative stress. Different forms of vascular calcification present different challenges. Medial vascular calcification increases arterial stiffness and cardiovascular risk without an obstruction of blood flow. Intimal vascular calcification contributes to plaque instability in atherosclerosis, leading to myocardial infarction or stroke. Additionally, genetic disorders such as CD73 deficiency (ACDC) further underscore the importance of phosphate metabolism and TNAP in disease progression.

Several therapeutic interventions have been explored with varying success. Bisphosphonates and phosphate binders have shown mixed results in both preclinical and clinical settings, while TNAP inhibitors, vitamin K supplementation, and anti-inflammatory strategies targeting cytokines like TNFα and IL-1β appear promising in reducing calcification burden or stabilizing plaques. The development and validation of appropriate in vitro and in vivo models, which distinguish between medial and intimal calcification, are critical for assessing these interventions. Biomechanical considerations are also essential; macrocalcifications tend to stabilize plaques and are detectable using standard imaging tools like CT and MRI, while microcalcifications are more dangerous, due to their role in plaque rupture, but are much harder to detect. Therapeutic efforts will need to dovetail with further development of advanced imaging modalities such as PET and OCT that may overcome resolution limitations.

Finally, the calcification paradox—the inverse relationship between vascular mineralization and bone mineral density—presents an additional layer of complexity. While bone and vascular tissues share some common regulators of mineralization, the cellular mechanisms diverge significantly. This divergence must be considered when developing therapies to avoid unintended effects on bone health.

### 4.2. Future Directions

Despite significant advances in understanding vascular calcification, there are still no approved therapeutics, and most current efforts focus on prevention rather than reversal. Pharmacological therapies capable of interrupting or dissolving previously formed calcification are difficult to develop and require a deeper understanding of the mechanisms driving calcification, including the distinction between medial and intimal calcification. Additionally, improving techniques to monitor mineralization changes during therapy may enable patient-specific interventions. A major challenge remains in mitigating off-target effects on bone health, as the calcification paradox is still not fully understood. Addressing these gaps through rigorous research will be critical to advancing viable therapeutics. By leveraging our growing knowledge of vascular calcification pathology, available models, and controllable mechanisms, we can move beyond prevention and toward transformative treatments that directly benefit patients.

Future therapeutic development in vascular calcification must prioritize specificity, efficacy, and safety. Approaches that target key regulators such as TNAP, components of the Wnt/BMP pathways, and vitamin K-dependent calcification inhibitors hold great promise. As many of these factors are shared with bone homeostasis, combination strategies that preserve or enhance bone health while inhibiting vascular mineralization must be considered. One of the most pressing needs is to understand whether vascular calcification can be reversed, and if so, how. While most current efforts aim at prevention, exploring the potential of reversing calcification, particularly through the activity of osteoclast-like cells within plaques, is a promising yet underexplored avenue. However, care must be taken, as reversing stable macrocalcifications could lead to the formation of unstable microcalcifications, potentially increasing the risk of acute cardiovascular events.

Preventive therapy remains an essential goal, especially for CKD patients who are at high risk of rapid calcification progression following the initiation of dialysis. These patients represent a clinically identifiable group that could be prioritized for early-phase therapeutic trials. Additionally, refining imaging techniques to monitor microcalcification and inflammation non-invasively could enable earlier detection and the better tracking of therapeutic outcomes, allowing for more tailored interventions. It is also critical that research models evolve to reflect the diverse pathologies of vascular calcification. Disease-specific models that differentiate between intimal and medial calcification and account for comorbidities like diabetes or genetic predisposition are necessary to ensure the clinical relevance of preclinical findings. Finally, therapeutic strategies must increasingly move toward personalized medicine, tailoring treatments based on the location, stage, and etiology of calcification to optimize outcomes while minimizing risks.

## Data Availability

No new data were created or analyzed in this study.

## Abbreviations

The following abbreviations are used in this manuscript:

CVD	Cardiovascular Disease
CKD	Chronic Kidney Disease
VSMC	Vascular Smooth Muscle Cells
EV	Extracellular Vesicles
LRP5/6	Low Density Lipoprotein Receptor-Related Protein
DVL	Disheveled Protein
GSK-3	Glycogen Synthase 3
APC	Adenomatous Polyposis Coli
RANKL	Receptor Activator Nuclear Factor- κB Ligand
OPG	Osteoprotegerin
VCAN	Versican
F-CPE	Full-Length Carboxypeptidase E
SOST	Sclorostin
BMP	Bone Morphogenic Protein
TGF-β	Transforming Growth Factor Beta
MGP	Matrix Gla Protein
LDL	Low-Density Lipoprotein
IL-1β	Interleukin β
ACDC	Arterial Calcification due to Deficiency of CD73
PPi	Pyrophosphate
Pi	Inorganic Phosphate
TNAP	Tissue Nonspecific Alkaline Phosphate
mTOR	Mammalian Target of Rapamycin
CT	Computed Tomography
P-407	Poloxamer 407
LPL	Lipoprotein Lipase
AGEs	Advanced Glycation End Products
RAGEs	Receptor for Advanced Glycation End Products
TNFα	Tumor Necrosis Factor-α
PPARγ	Peroxisome Proliferator-Activated Receptor Gamma
CANTOS	Canakinumab Anti-inflammatory Thrombosis Outcome Study
MRI	Magnetic Resonance Imaging
PET	Positron Emission Tomography
^18^F-NaF	^18^F-sodium fluoride
^18^F-DG	^18^F-fluorodeoxyglucose
OCT	Optical Coherence Tomography
